# Clarithromycin and Glipizide Drug-drug Interaction Leading to Refractory Hypoglycemia

**DOI:** 10.7759/cureus.4800

**Published:** 2019-06-02

**Authors:** Xu Cong Ruan, Poh Yong Tan, Yuyang Tan

**Affiliations:** 1 Internal Medicine, Singapore General Hospital, Singapore, SGP; 2 Internal Medicine, Singapore General Hospital, SingHealth, Singapore, SGP

**Keywords:** diabetes, drug interactions, hypoglycemia

## Abstract

A 70-year-old end-stage renal disease patient was admitted for refractory hypoglycemia secondary to drug-drug interaction between clarithromycin and glipizide. We discussed the mechanism of antimicrobial and sulfonylurea interactions as well as the importance of understanding these interactions in the primary care setting to reduce medication-related hospitalizations.

## Introduction

Hypoglycemia is a common problem faced by many patients with diabetes mellitus, especially when they are treated with glucose-lowering medication. Causes of hypoglycemia include drugs, sepsis, organ failure, insulinoma, prescribing/dispensing errors and post bariatric surgery [1]. Refractory hypoglycemia is defined as capillary blood glucose (CBG) persistently less than 80 mg/dL despite initial management with dextrose 50%. Prolonged hypoglycemia can increase neuronal cell death and this is especially prominent in high risk individuals such as those who are elderly, with chronic kidney disease, and suffer from polypharmacy [2]. We present a case of a patient who had refractory hypoglycemia attributed to antimicrobial and sulfonylurea interaction on background of end-stage renal disease on hemodialysis.

## Case presentation

A 70-year-old Chinese female was admitted to a tertiary hospital for nonspecific symptoms of non-vertiginous dizziness with lethargy for two days. She has a history of diabetes of more than 20 years with recent tight glycemic control (HbA1c 5.4%). She also has end-stage renal failure on hemodialysis three times a week (eGFR < 15 ml/min). Her other past medical history includes hypertension, ischemic heart disease and metastatic breast cancer on exemestane, with complete response. Her medications include aspirin 100 mg OM, amlodipine 10 mg OM, losartan 75 mg OM, exemestane 25 mg OM, atorvastatin 40 mg ON, ferrous fumarate 200 mg BD, omeprazole 20 mg OM, cholecalciferol 1000 units OM, recormon 4000 units thrice a week, iron injection 100 mg every fortnight and calcium carbonate 1.25 g pre-lunch and pre-dinner. Her CBG measured at home was 70-90 mg/dL in the month prior to admission, despite taking regular meals. She has no hypoglycemia symptoms at baseline.

One week prior to admission, she visited her family physician for cough and fever lasting for a week, and was prescribed a course of clarithromycin for five days. There was no chest radiograph performed during this visit. Her last dose of clarithromycin was a day prior to admission.

**Figure 1:**
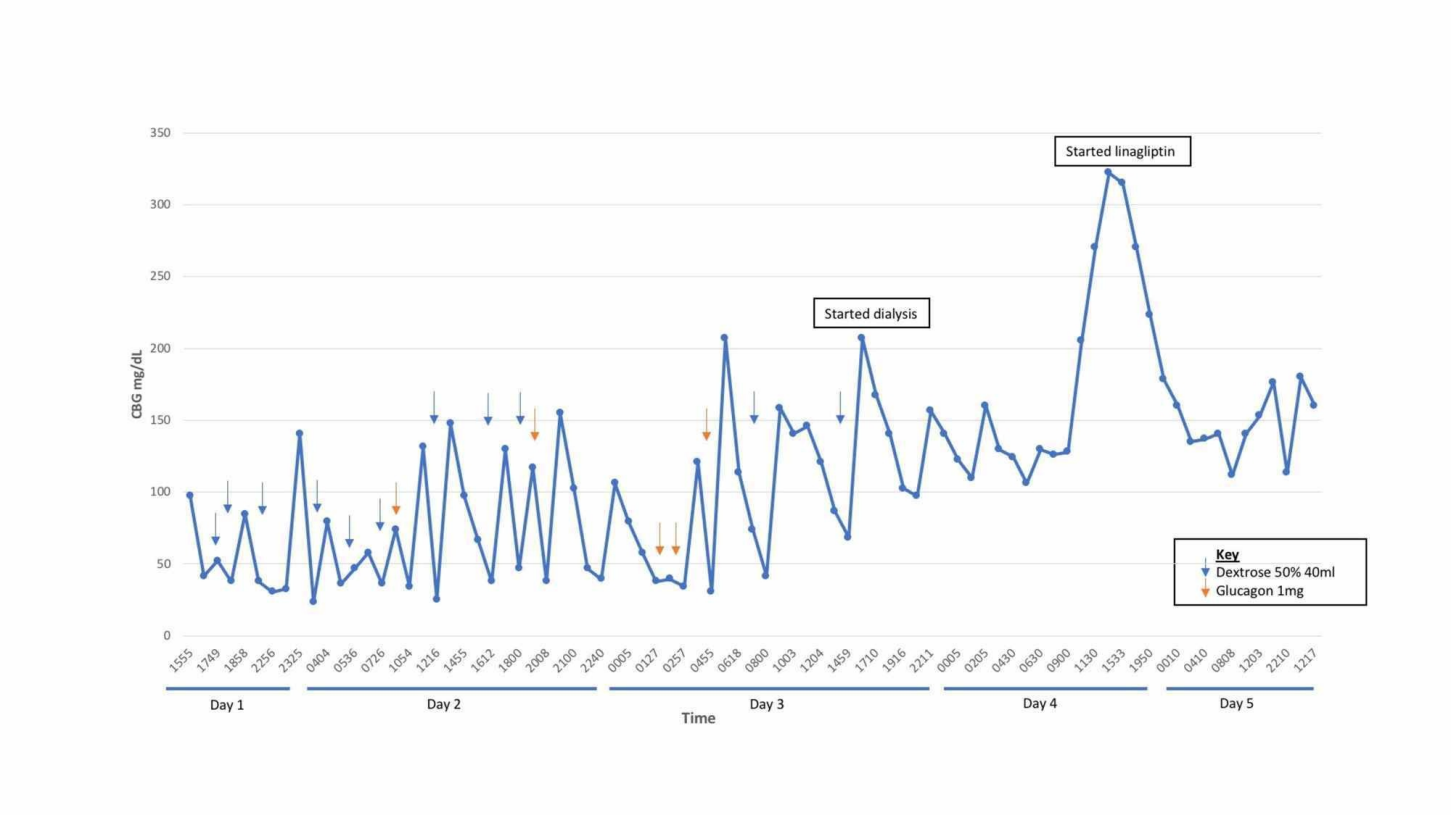
CBG trend of patient during inpatient stay and with illustrations of interventions provided, dextrose 50% 40 ml (blue arrows) and glucagon 1 mg (orange arrows). CBG: Capillary blood glucose

On the day of admission, in view of her symptoms of dizziness and lethargy, she checked her CBG and noted that it was 50 mg/dL, which did not improve despite her taking additional meals and dextrose drinks. Beyond lethargy and giddiness, she did not have any other symptoms suggestive of neuroglycopenia. During her inpatient stay, her CBG was trended (Figure 1) and she was persistently hypoglycemic despite 11 boluses of 40 ml 50% dextrose, five intravenous boluses of 1 mg glucagon and a maintenance 10% dextrose drip. C-peptide was 23 UG/L (0.78-5.19 UG/L) and insulin level 34.5 mU/L (1.0-30.0 mU/L). This suggested increased insulin secretion from glipizide ingestion. We referred the patient to the endocrinology team. Their impression was that the refractory hypoglycemia was attributed to glipizide and clarithromycin drug-drug interaction in a renally impaired patient. Interestingly, her CBG improved to 120 mg/dL after hemodialysis, which likely assisted in removing serum glipizide. In view of her tight CBG control, glipizide was discontinued and she was placed on linagliptin, which was less likely to cause hypoglycemia.

## Discussion

This case illustrates how an elderly patient with end-stage renal failure on glucose-lowering medication can be predisposed to severe refractory hypoglycemia. In view of her advanced age, she may not illustrate early symptoms of hypoglycemia. In addition, her body was also less able to exert counter-regulatory response to hypoglycemia. In view of her renal impairment, there was progressive reduction in her insulin requirements through decreased renal clearance, degradation of insulin in peripheral tissues and renal gluconeogenesis [3]. Therefore, when her glucose-lowering medication was not titrated accordingly, it might account for the progressive decrease in her CBG trend prior to admission.

The main adverse effect of sulfonylurea is hypoglycaemia, which can be aggravated through the addition of hepatic cytochrome P450 inhibitors, such as antimicrobial agents [4,5]. In fact, it is estimated that 12.3% of all hypoglycemic events in patient prescribed sulfonylureas are associated with antimicrobial use, especially fluoroquinolones, macrolides, sulfamethoxazole-trimethoprim and azoles (Table 1) [4].

**Table 1:** Common antimicrobial interactions with sulfonylurea leading to hypoglycemia.

Drug	Pharmacodynamic Mechanism	Pharmacokinetic Mechanism	
		Distribution	Metabolism
Macrolides, e.g., clarithromycin	-	Macrolides displace sulfonylurea from its protein bound state, leading to increased serum-free levels causing hypoglycemia	Macrolides are P-glycoprotein inhibitors reducing the efflux of sulfonylurea from enterocytes, leading to increased serum levels precipitating hypoglycemia
Azoles, e.g., fluconazole, voriconazole	-	-	Azoles inhibit CYP2C9, therefore increasing serum sulfonylurea levels
Fluoroquinolones, e.g., moxifloxacin, ciprofloxacin, levofloxacin	Fluoroquinolones augment sulfonylurea in its inhibition of ATP K+ channels in pancreatic B-cells leading to earlier depolarization initiating insulin secretion	-	-
Sulfamethoxazole-trimethoprim	-	-	Sulfamethoxazole-trimethoprim inhibit CYP2C9, therefore increasing serum sulfonylurea levels

There are multiple postulations to explain this interaction. In addition to affecting hepatic metabolism, these agents also influence the distribution and elimination of sulfonylureas. Sulfonylureas are 90-99% protein bound, whereas macrolides such as clarithromycin are 40-70% protein bound [6]. The addition of macrolides can lead to the displacement of glipizide, leading to increased free serum glipizide in the body which exacerbates hypoglycemia. In addition, macrolides are P-glycoprotein inhibitors and may reduce the removal of sulfonylureas from the systemic circulation [4]. Lilja et al. illustrated that serum glibenclamide levels in volunteers were elevated by 24% after taking clarithromycin [7].

Macrolides are well known CYP3A4 inhibitors. Unfortunately, glipizide is mostly cleared by CYP2C9. The inhibition of CYP3A4 by macrolides has minimum effect on the increase in serum glipizide levels [6].

For antifungals such as fluconazole and voriconazole, as well as sulfamethoxazole-trimethoprim, they are known to be CYP2C9 inhibitors. Since sulfonylureas are cleared by CYP2C9, their serum concentrations increase with the concomitant use of these medication, leading to hypoglycemia from the excessive release of insulin [5,8].

Fluoroquinolones, on the other hand, augments the pharmacodynamics action of sulfonylurea through inhibiting adenosine triphosphate-potassium channels (ATP-K+) in pancreatic B-cells, thereby leading to earlier depolarization, increasing insulin release causing hypoglycemia [9].

For our patient, her hypoglycemia was persistent despite multiple boluses of high concentration dextrose and intravenous glucagon. Glucagon was employed to counter hypoglycemia as it simulates glycogenolysis in the liver [10]. It is used as a salvage therapy for those with severe refractory hypoglycemia not responding to intravenous dextrose boluses.

Interesting, while our patient did not respond to conventional therapy, her CBG improved immediately after dialysis. While we understand that glipizide is non-dialyzable when protein bound, it is likely that the clearance of unbound glipizide was made possible through the dialysis semipermeable membrane in view of its smaller molecular size [11].

In retrospect, for patients with renal impairment who illustrates symptoms of upper respiratory tract infection, it is prudent to delineate if her infection is viral or bacterial in origin to minimize the over-prescription antimicrobial agents. If indeed she does have pneumonia secondary to bacterial infection, a combination of amoxicillin/clavulanic acid with doxycycline would have been preferred to reduce the chance of drug-drug interactions.

## Conclusions

Drug-drug interactions and overtly tight glycemic control often lead to hypoglycemia in patients with diabetes mellitus. Hospitalization from hypoglycemia is associated with poor outcomes, especially in older patients, leading to death and neuroglycopenia. It is important to prevent polypharmacy in these individuals to reduce hospital admission rates. Interventions to enhance physicians’ knowledge of potential drug-drug interactions and judicious use of antimicrobials in this group of patients will facilitate care and reduce readmissions. It is also important to assess the glycemic control in individual patients based on their age and comorbidities such as liver and renal function to decide on the level of CBG control that is most appropriate.
